# Takayasu Arteritis With Antiphosphatidylserine/Prothrombin Antibody-Positive Antiphospholipid Syndrome: Case Report and Literature Review

**DOI:** 10.1097/MD.0000000000002345

**Published:** 2015-12-28

**Authors:** Shoichi Fukui, Shogo Hirota, Naoki Iwamoto, Hiroki Karata, Atsushi Kawakami

**Affiliations:** From the Department of Immunology and Rheumatology, Nagasaki University Graduate School of Biomedical Sciences, 1-7-1 Sakamoto, Nagasaki (SF, SH, NI, AK); and Department of Pathology, Nagasaki University Hospital, Nagasaki, Japan (HK).

## Abstract

A relationship between Takayasu arteritis (TA) and positive antiphospholipid antibody states has been pointed out, but patients with TA complicated with antiphospholipid antibody syndrome (APS) are rare. Here we report the case of a 17-year-old Japanese man diagnosed with TA based on pulselessness of the left brachial artery, discrepancy of blood pressure between the upper extremities, and arterial wall thickening and narrowing of artery in contrast computed tomography. He was also diagnosed with provisional APS based on a pulmonary infarction without narrowing of the pulmonary artery and positive antiphosphatidylserine/prothrombin antibody. The patient also had concurrent Crohn's disease (CD) based on histopathological findings, which may have been associated with TA. We started high-dose corticosteroid therapy and anticoagulation therapy, and his symptoms including fever, dizziness, chest pain, and lower-right uncomfortable abdomen improved.

We reviewed 9 cases of TA with APS including our patient by conducting a PubMed search. Based on past reports, we considered the relationship among TA, APS, and CD.

Clinicians should bear in mind that many etiologies can exist in 1 patient, and differential diagnoses are essential.

## INTRODUCTION

Takayasu arteritis (TA) is a rare large-vessel vasculitis variant that affects predominantly young women.^1^ TA affects the aorta and its main branches^2^ and the pulmonary arteries.^3^ TA is commonly seen in Japan, South East Asia, India, and Mexico.^4^ It was reported that 150 new TA cases occur each year in Japan,^5^ whereas the reported incidence of TA in Olmsted County, Minnesota, United States, was 2.6 new cases per year per million population.^6^

Antiphospholipid syndrome (APS) is characterized by obstetric and thrombotic complications in the presence of antiphospholipid antibodies, which consist of anticardiolipin antibody (aCL), lupus anticoagulant (LA), and anti-β2 glycoprotein I (aβ2GP I). In addition, antiphosphatidylserine/prothrombin antibody (aPS/PT) was revealed to be associated with APS.^7^ An association between TA and APS has rarely been described. Here we report a case of TA associated with APS with positive aPS/PT.

## Case Presentation

A 17-year-old Japanese man was admitted to our hospital complaining of body weight loss and fever. The weight loss began 6 months prior to this admission, amounting to a 15 kg reduction as of the admission. Four months prior to this admission, he began feeling general fatigue and dizziness when he changed the position of his head. Three months prior to the admission, he experienced instant chest pain on the left side when he breathed to the maximal inspiratory level, which had nothing to do with the time of day, and his lower right abdomen felt uncomfortable (which was a heavy feeling but not pain) in the lower right abdomen that had nothing to do with his meal intake. He did not have any symptoms such as changes in bowel habits, diarrhea, constipation, and hematochezia. Two weeks before his admission, the fever emerged. He did not have any notable medical history.

His physical examination on admission revealed the following: body temperature 37.5°C; blood pressure, right arm 112/68 mm Hg, left arm could not be measured; pulse rate 90/min; and respiratory rate 16/min. Auscultation of the chest showed no heart murmur or crackles. Pulses of the left brachial and radial arteries were not palpable. There were no skin eruptions.

His laboratory test results were as follows: white blood cell (WBC) count 10,300/μL (neutrophils 75%, lymphocytes 22%, monocytes 3%, eosinophils 0%, and basophils 0%); hemoglobin 10.8 g/dL; platelets 466,000/μL; C-reactive protein (CRP) 16.6 mg/dL (normal range <0.3 mg/dL); erythrocyte sedimentation rate (ESR) 87 mm/h (normal range 1–10 mm/h); serum creatinine (Cr) 0.56 mg/dL (normal values <1.0 mg/dL). He had a prolonged activated partial thromboplastin time (aPTT) (60.3 s, normal range: 24.3–36.0 s), and elevated D-dimer (1.2 μg/mL, normal values <1.0 μg/mL). All of the following were negative: aCL (by enzyme-linked immunosorbent assay [ELISA]), LA (by diluted Russell's viper venom time test), and aβ2GP I (by ELISA). Antiphosphatidylserine/prothrombin antibody (aPS/PT) (IgG, by ELISA) was positive (18 U/mL, normal values <10 U/mL). Antinuclear antibody was negative. Human leukocyte antigen (HLA) typing was ^∗^B15:0101/^∗^B52:0101. A urinalysis did not show any remarkable data.

**FIGURE 1:**
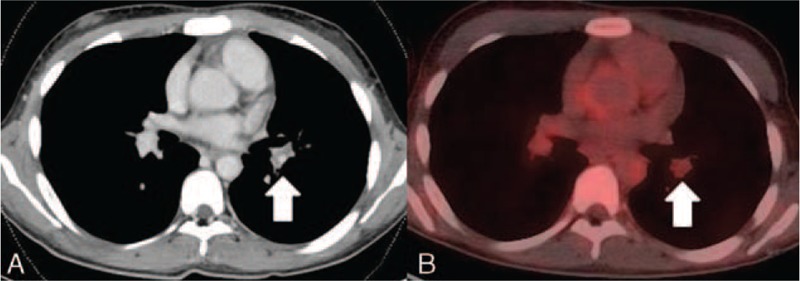
(A) Defect of contrast in the pulmonary artery without narrowing of the pulmonary artery, which suggested thrombosis (arrow). (B) PET showed no FDG accumulation in the wall of the same lesion of the pulmonary artery (arrow). FDG = 18F- fluoro-2-deoxy-D-glucose, PET = positron emission tomography.

Contrast computed tomography (CT) showed arterial wall thickening of the ascending and descending aorta and narrowing of the left subclavian artery. Positron emission tomography (PET) showed 18F- fluoro-2-deoxy-D-glucose (FDG) accumulation in the wall of the left subclavian artery. A defect of contrast in the pulmonary artery without narrowing suggested pulmonary thrombosis (Fig. 1A). PET showed no FDG accumulation in the wall of the same lesion of the pulmonary artery (Fig. 1B).

**FIGURE 2:**
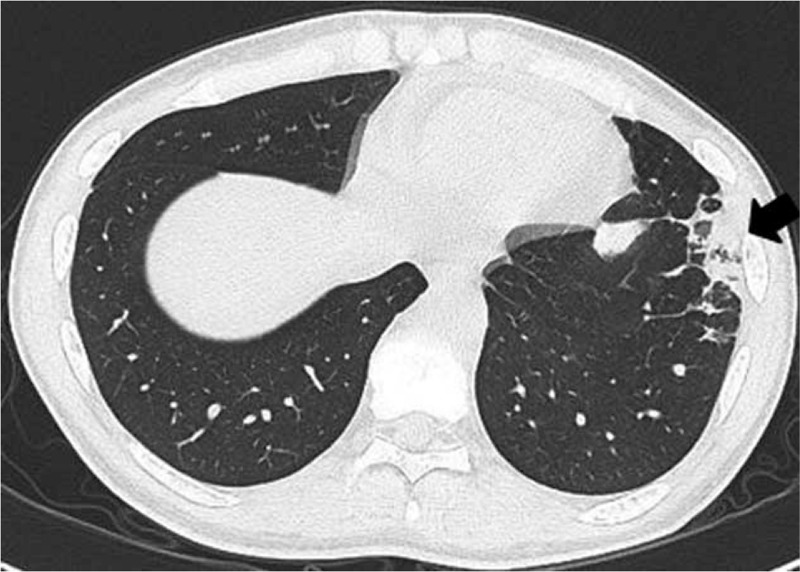
CT showed subpleural wedge-shaped consolidation in the area that was perfused by the obstructed pulmonary artery (arrow).

**FIGURE 3:**
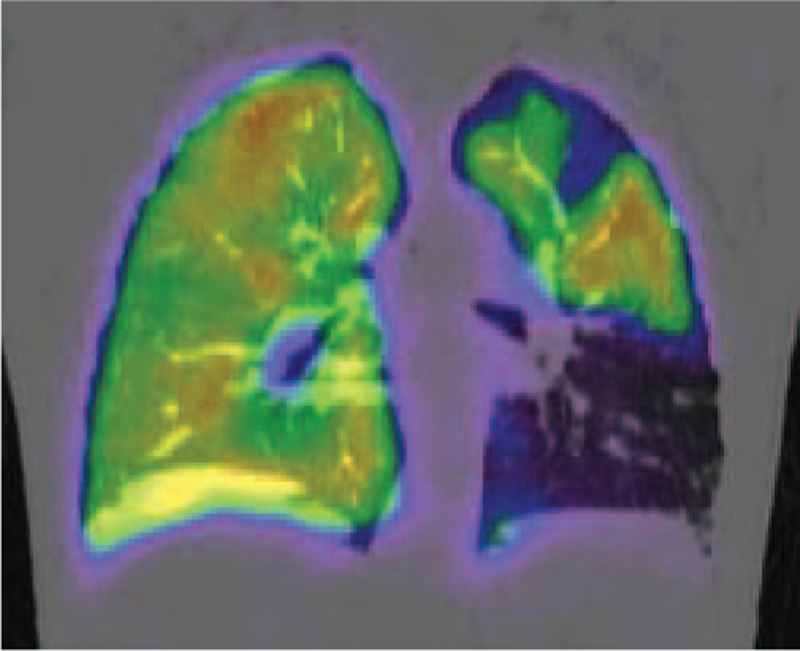
Ventilation perfusion lung scintigraphy showed a mismatch of perfusion and air in the left lung.

**FIGURE 4:**
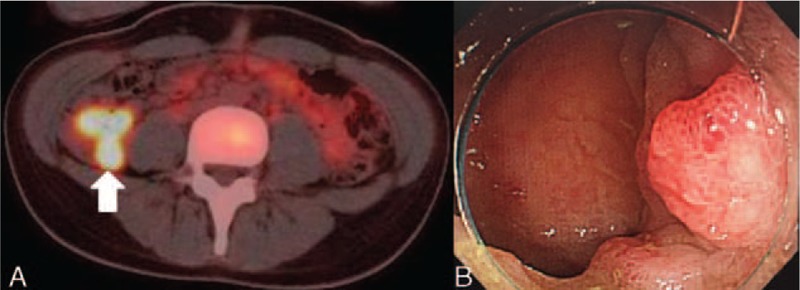
(A) PET showed FDG collection in the ileocecum. (B) Colonoscopy showed redness of mucus of the ileocecum. FDG = 18F- fluoro-2-deoxy-D-glucose, PET = positron emission tomography.

**FIGURE 5:**
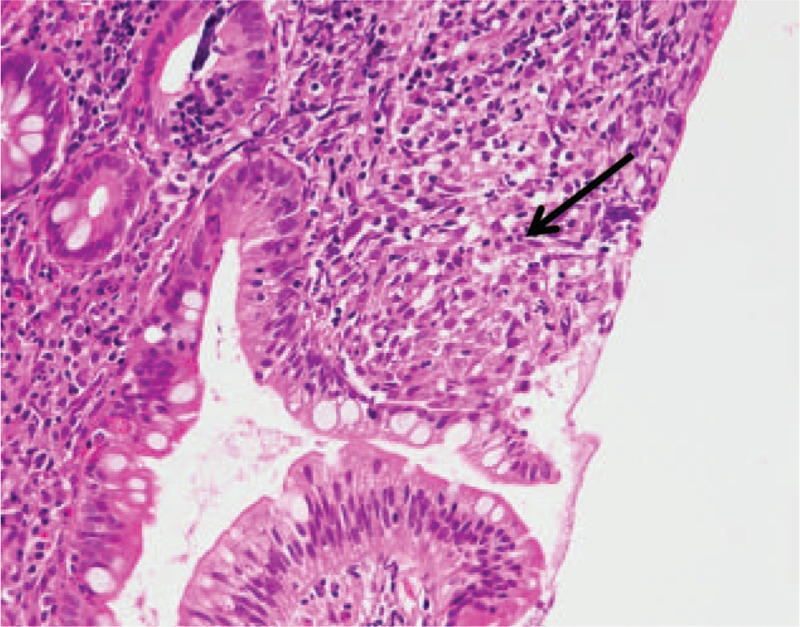
The biopsy specimen of the ileocecum shows a noncaseating epithelioid granuloma (arrow).

In the patient's left lung, subpleural wedge-shaped consolidation was observed in the area that was perfused by the obstructed pulmonary artery (Fig. 2). Ventilation perfusion lung scintigraphy showed a mismatch of perfusion and air in the left lung (Fig. 3). PET also showed FDG collection in the ileocecum (Fig. 4A). Colonoscopy showed redness of mucus of the ileocecum (Fig. 4B). The biopsy specimen of the ileocecum showed a noncaseating epithelioid granuloma (Fig. 5).

The patient's findings fulfilled the following criteria issued by the American College of Rheumatology 1990 for the classification of TA^8^: age at disease onset ≤40 years, decreased brachial artery pulse, blood pressure difference >10 mm Hg, and arteriogram abnormality, and we therefore diagnosed TA. We suspected that the dizziness experienced by the patient when he changed the position of his head was induced by subclavian steal blood syndrome, because his left subclavian artery was severely narrowed. Because the patient also had a pulmonary embolism, an infarction without narrowing of the pulmonary artery, and positive aPS/PT, we diagnosed provisional APS pending a confirmatory lab result. In addition, based on the histopathological findings of the ileocecum, we also diagnosed Crohn's disease (CD).

We started 50 mg prednisolone per day and anticoagulant therapy by warfarin. The patient's fever and chest pain when he breathed then disappeared quickly. His CRP level and ESR declined. One month after the initiation of treatment, CT showed a partial recanalization of the previously occluded left subclavian artery. Sixteen weeks after the initiation of treatment, antiphosphatidylserine antibody was also positive (12 U/mL). We made a diagnosis of provisional APS with positive aPS/PT. A new pulmonary embolism did not occur during the subsequent 1 year of follow-up. His left lower abdominal discomfort never emerged again.

## DISCUSSION

We have reported a case of TA associated with APS with positive aPS/PT. Our patient's case had 2 characteristic features: TA complicated with APS, and CD. Regarding the complication of APS, 2 problems hamper the diagnosis of APS. One is that the classification criteria for definite APS (ie, the Sapporo criteria)^9^ do not include aPS/PT as a specific antibody for APS. It is now acknowledged that APS can exist in the presence of autoantibodies other than aCL, LA, and aβ2GPI. For example, vimentin, annexin A5, and annexin 2 are known as new autoantigens in the APS.^10^ In addition, isotype is important. For example, IgA aβ2GP and IgA aCL are known.^11^ One of the “new” autoantibodies in APS is aPS/PT, and some authors have reported the usefulness of aPS/PT for the diagnosis of APS. For example, Atsumi et al reported that aCL and IgG aPS/PT have similar diagnostic value for APS.^12^ Khogeer et al reported a significant association between IgG antiphosphatidylserine antibodies and APS, especially when they were used to diagnosis clinical cases with other negative aPL tests.^13^ Based in part on these reports, we concluded that our patient had APS.

We suspect that the presence of aPS/PT is independent of TA in nature. However, it is difficult to deny that aPS/PT may become positive in cases of TA without APS. Regarding other autoantibodies in APS, Misra et al reported that of 34 patients with TA, 14 (41%) had an increased IgG aCL level and none of the 14 had features suggestive of APS.^14^ Jordan et al reported that of 22 patients with TA, 10 (45%) had persistently positive antiphospholipid antibodies or concurrent APS, and the TA patients with positive antiphospholipid antibodies had more vascular complications.^15^ Tripathy et al reported that of 66 patients with TA, 24 (36%) had positive antiannexin V antibodies.^16^ However, we could not find past findings or reports regarding this problem between TA and aPS/PT. To resolve this problem, the prevalence of aPS/PT in TA patients without APS should be determined.

Another issue is whether vasculitis exists in the obstructed pulmonary artery in TA. The most important point in the diagnosis of APS is the determination of whether the vascular obstruction is due to vasculitis. This is pointed out in the Sapporo criteria.^9^ Our patient had a pulmonary embolism without vasculitis of the pulmonary artery as shown by imaging, particularly PET, which showed no FDG accumulation in the wall of the obstructed lesion of the pulmonary artery. Indeed, without a biopsy it would have been difficult to demonstrate the absence of vasculitis of his pulmonary artery. However, contrast CT also showed no narrowing or wall thickening of the pulmonary artery, and thus we suspect that the obstructed pulmonary artery did not have vasculitis.

We searched for the existing case reports of TA associated with APS by conducting a PubMed search (http://www.ncbi.nlm.nih.gov/pubmed) without a date, using the search terms “Takayasu arteritis” and “antiphospholipid antibody syndrome.” We found 8 cases of TA with APS other than our patient (Table 1). We found 1 definite APS (classified by Sapporo criteria) patient and 2 other patients with clinical and serological features of APS (insufficient to satisfy the Sapporo criteria) in a report by Jordan et al, but the detailed clinical history of these 3 patients was not available and thus we excluded them. We also excluded patients with solely positive antiphospholipid antibodies without thrombotic events (ie, arterial thrombosis, venous thrombosis, or pregnancy loss).

**TABLE 1:**
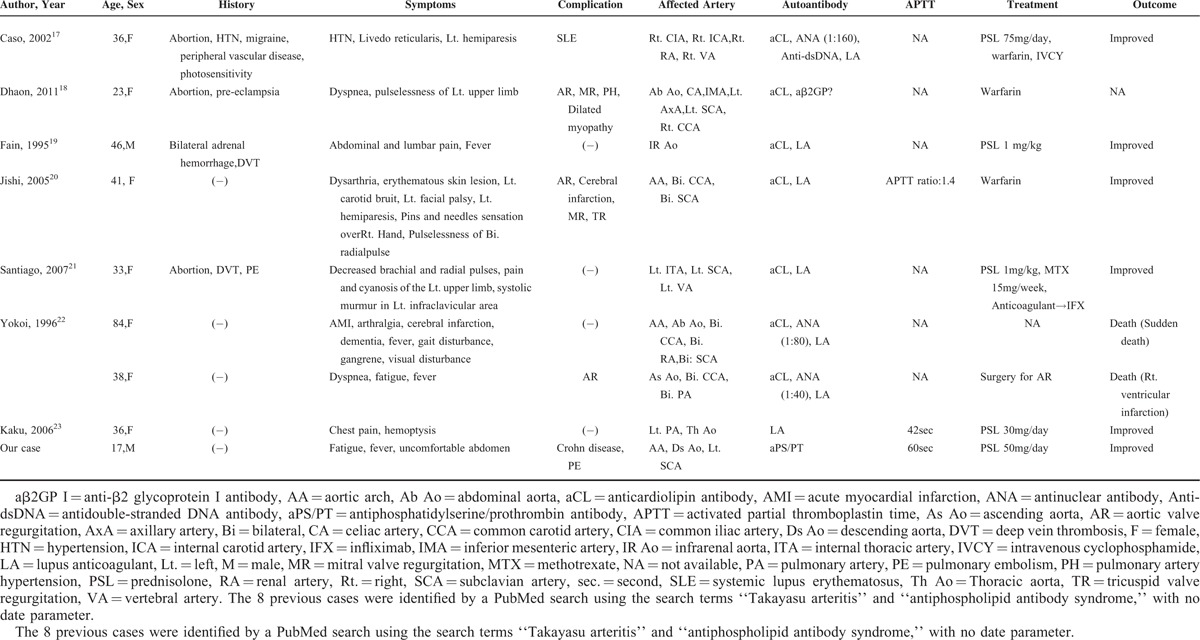
Nine Cases of Takayasu Arteritis (TA) With Antiphospholipid Syndrome (APS)

As shown in the table, of the 9 cases (including our patient), 7 patients had aCL^17–22^ and 7 patients had LA.^17,19–23^ Only 1 patient had positive aβ2GP I.^18^ aPS/PT was not checked in any of the cases shown in the table except for our patient. To our knowledge, this is the first case report of a TA patient with APS who had positive aPS/PT. Two of the 9 patients (including our case) were men.^19^ Three patients had undergone an abortion.^17,18,21^ Deep vein thrombosis was seen in 2 patients.^19,21^ One patient's case was complicated with systemic lupus erythematosus (SLE).^17^ Although in SLE cases, small-vessel involvement is the most frequent, medium- and large-vessel vasculitis may present with visceral affection.^24^ It was therefore difficult to distinguish TA from large-vessel vasculitis of SLE in the case reported by Caso et al.

Our patient also had CD. Reny et al reported that 9% of 44 TA patients had CD.^25^ Although the pathogeneses of both TA and CD are unclear, some similarities have been pointed out. The fact that both TA and CD show granulomatous changes histopathologically^26,27^ may affect this high concurrence ratio. In addition, tumor necrosis factor-alpha (TNF-α) monoclonal antibody is effective for both TA^28^ and CD,^29^ which may suggest the presence of a common inflammatory pathway in both diseases.

On the other hand, Kraiem et al reported that some antiphospholipid antibodies were positive in 54.2% of 48 CD patients.^30^ Sipeki et al reported that of 264 CD patients, aPS/PT was positive in 20.4% (any 1 of IgG, IgM, or IgA)^31^ and that 14.6% of the CD patients without events of thrombosis had positive aPS/PT, whereas 50.0%, 36.4%, or 40.0% of the CD patients with events of arterial thrombosis, venous thrombosis, or pregnancy loss had positive aPS/PT, respectively.

Bacterial translocation (as one of the causes of the production of autoantibodies) is thought to induce autoantibodies in inflammatory bowel disease.^32^ Similarly, bacterial translocation may be associated with the production of aPS/PT in CD patients. Although it is not general practice to measure antiphospholipid antibodies when diagnosing CD, there are many more CD patients with positive antiphospholipid antibodies than we expected. Measuring antiphospholipid antibodies in CD patients may be important to determine the prognosis regarding thrombosis.

Although the relationships among TA, APS, and CD in our patient are unclear, autoantibodies, the formation of granuloma, and bacterial involvement may have been associated with his case's etiology.

In conclusion, we have reported the case of a young adult patient with TA complicated with APS, which is very rare. Clinicians should bear in mind that many etiologies can exist in 1 patient and that differential diagnoses are essential. Further studies of TA and APS regarding clinical and prognostic differences are needed.

## CONSENT

Written informed consent was obtained from the patient for the publication of this case report. A copy of the written consent is available for review by the editor of this journal.

## References

[1] Lupi-Herrera E Sanchez-Torres G Marcushamer J Takayasu's arteritis. Clinical study of 107 cases. *Am Heart J* 1977; 93:94–103. 12655 10.1016/s0002-8703(77)80178-6

[2] Hata A Noda M Moriwaki R Angiographic findings of Takayasu arteritis: new classification. *Int J Cardiol* 1996; 54 (Suppl:) S155–S163. 9119518 10.1016/s0167-5273(96)02813-6

[3] Pulmonary pulseless disease: pulmonary involvement in so-called Takayasu's disease, Clinical Conference in Cardiology from the third medical division, Kyoto University Hospital, Kyoto, Japan Chest, 73, 1978, 651–657. 25748

[4] Johnston SL Lock RJ Gompels MM Takayasu arteritis: a review. *J Clin Pathol* 2002; 55:481–486. 12101189 10.1136/jcp.55.7.481 PMC1769710

[5] Koide K Takayasu arteritis in Japan. *Heart Vessels Suppl* 1992; 7:48–54. 1360971 10.1007/BF01744544

[6] Hall S Barr W Lie JT Takayasu arteritis: a study of 32 North American patients. *Medicine (Baltimore)* 1985; 64:89–99. 2858047

[7] Atsumi T Ieko M Bertolaccini ML Association of autoantibodies against the phosphatidylserine–prothrombin complex with manifestations of the antiphospholipid syndrome and with the presence of lupus anticoagulant. *Arthritis Rheum* 2000; 43:1982–1993. 11014348 10.1002/1529-0131(200009)43:9<1982::AID-ANR9>3.0.CO;2-2

[8] Arend WP Michel BA Bloch DA The American College of Rheumatology 1990 criteria for the classification of Takayasu arteritis. *Arthritis Rheum* 1990; 33:1129–1134. 1975175 10.1002/art.1780330811

[9] Miyakis S Lockshin MD Atsumi T International consensus statement on an update of the classification criteria for definite antiphospholipid syndrome (APS). *J Thromb Haemost* 2006; 4:295–306. 16420554 10.1111/j.1538-7836.2006.01753.x

[10] Alessandri C Conti F Pendolino M New autoantigens in the antiphospholipid syndrome. *Autoimmun Rev* 2011; 10:609–616. 21545849 10.1016/j.autrev.2011.04.011

[11] Rodriguez-Garcia V Ioannou Y Fernandez-Nebro A Examining the prevalence of non-criteria anti-phospholipid antibodies in patients with anti-phospholipid syndrome: a systematic review. *Rheumatology (Oxford)* 2015; 54:2042–2050. 26152548 10.1093/rheumatology/kev226

[12] Atsumi T Koike T Antiprothrombin antibody: why do we need more assays? *Lupus* 2010; 19:436–439. 20353984 10.1177/0961203310361487

[13] Khogeer H Alfattani A Al Kaff M Antiphosphatidylserine antibodies as diagnostic indicators of antiphospholipid syndrome. *Lupus* 2015; 24:186–190. 25253571 10.1177/0961203314552462

[14] Misra R Aggarwal A Chag M Raised anticardiolipin antibodies in Takayasu's arteritis. *Lancet* 1994; 343:1644–1645. 7911954 10.1016/s0140-6736(94)93103-8

[15] Jordan NP Bezanahary H D’Cruz DP Increased risk of vascular complications in Takayasu's arteritis patients with positive lupus anticoagulant. *Scand J Rheumatol* 2015; 44:211–214. 25438797 10.3109/03009742.2014.964305

[16] Tripathy NK Sinha N Nityanand S Anti-annexin V antibodies in Takayasu's arteritis: prevalence and relationship with disease activity. *Clin Exp Immunol* 2003; 134:360–364. 14616799 10.1046/j.1365-2249.2003.02282.x PMC1808857

[17] Caso V Paciaroni M Parnetti L Stroke related to carotid artery dissection in a young patient with Takayasu arteritis, systemic lupus erythematosus and antiphospholipid antibody syndrome. *Cerebrovasc Dis* 2002; 13:67–69. 11810014 10.1159/000047749

[18] Dhaon P Das SK Saran RK Is aorto-arteritis a manifestation of primary antiphospholipid antibody syndrome? *Lupus* 2011; 20:1554–1556. 21846694 10.1177/0961203311412414

[19] Fain O Mathieu E Seror O Aortitis: a new manifestation of primary antiphospholipid syndrome. *Br J Rheumatol* 1995; 34:686–687. 7670792 10.1093/rheumatology/34.7.686

[20] Jishi AA Krishnan PR Almawi WY Takayasu arteritis with high titre of antiphospholipid antibodies and MTHFR polymorphism. *J Thromb Thrombolysis* 2005; 20:47–50. 16133896 10.1007/s11239-005-2459-2

[21] Santiago MB Paz O Rare association of antiphospholipid syndrome and Takayasu arteritis. *Clin Rheumatol* 2007; 26:821–822. 16575491 10.1007/s10067-006-0277-3

[22] Yokoi K Hosoi E Akaike M Takayasu's arteritis associated with antiphospholipid antibodies. Report of two cases. *Angiology* 1996; 47:315–319. 8638879 10.1177/000331979604700317

[23] Kaku B Higuchi T Kanaya H Usefulness of fluorine-18-fluorodeoxyglucose positron emission tomography in a patient with Takayasu's arteritis associated with antiphospholipid syndrome. *Int Heart J* 2006; 47:311–317. 16607057 10.1536/ihj.47.311

[24] Barile-Fabris L Hernandez-Cabrera MF Barragan-Garfias JA Vasculitis in systemic lupus erythematosus. *Curr Rheumatol Rep* 2014; 16:440. 25023725 10.1007/s11926-014-0440-9

[25] Reny JL Paul JF Lefebvre C Association of Takayasu's arteritis and Crohn's disease. Results of a study on 44 Takayasu patients and review of the literature. *Ann Med Interne (Paris)* 2003; 154:85–90. 12746644

[26] Nasu T Takayasu's truncoarteritis. Pulseless disease or aortitis syndrome. *Acta Pathol Jpn* 1982; 32 (Suppl 1:) 117–131. 6139930

[27] Magro F Langner C Driessen A European consensus on the histopathology of inflammatory bowel disease. *J Crohns Colitis* 2013; 7:827–851. 23870728 10.1016/j.crohns.2013.06.001

[28] Mekinian A Neel A Sibilia J Efficacy and tolerance of infliximab in refractory Takayasu arteritis: French multicentre study. *Rheumatology (Oxford)* 2012; 51:882–886. 22223706 10.1093/rheumatology/ker380

[29] Targan SR Hanauer SB van Deventer SJ A short-term study of chimeric monoclonal antibody cA2 to tumor necrosis factor alpha for Crohn's disease. Crohn's Disease cA2 Study Group. *N Engl J Med* 1997; 337:1029–1035. 9321530 10.1056/NEJM199710093371502

[30] Kraiem I Hadhri S Rejeb MB Antiphospholipid antibodies and procoagulant profile in Tunisians with inflammatory bowel diseases. *Clin Appl Thromb Hemost* 2015; [Epub ahead of print]. 10.1177/1076029615581364 25878173

[31] Sipeki N Davida L Palyu E Prevalence, significance and predictive value of antiphospholipid antibodies in Crohn's disease. *World J Gastroenterol* 2015; 21:6952–6964. 26078573 10.3748/wjg.v21.i22.6952 PMC4462737

[32] Papp M Lakatos PL Serological studies in inflammatory bowel disease: how important are they? *Curr Opin Gastroenterol* 2014; 30:359–364. 24811052 10.1097/MOG.0000000000000076

